# Neural Survival Clustering: Non-parametric mixture of neural networks for survival clustering

**Published:** 2022

**Authors:** Vincent Jeanselme, Brian Tom, Jessica Barrett

**Affiliations:** MRC Biostatistics Unit, University of Cambridge, UK

## Abstract

Survival analysis involves the modelling of the times to event. Proposed neural network approaches maximise the predictive performance of traditional survival models at the cost of their interpretability. This impairs their applicability in high stake domains such as medicine. Providing insights into the survival distributions would tackle this issue and advance the medical understanding of diseases. This paper approaches survival analysis as a mixture of neural baselines whereby different baseline cumulative hazard functions are modelled using positive and monotone neural networks. The efficiency of the solution is demonstrated on three datasets while enabling the discovery of new survival phenotypes.

## 1 Introduction

Predicting the risk of a medical event is essential for clinical screening, prioritisation and intervention. Survival analysis has been used in the literature to model the time to an event such as death or the appearance of symptoms. This analysis differs from standard regression problems as it leverages information from patients for whom the outcome of interest was unobserved. Though the event of interest has not occurred during the follow-up period for these patients, their *censored* data still contribute to the likelihood through the knowledge that their times to the event must be later than their observed right-censoring times.

Extensive research has developed likelihood-based survival models which allow for censored observations. Approaches have limited the complexity of the model's likelihood. In the statistical literature, parametric models have been used when the survival functional form is known or for computational tractability and interpretability. Semi-parametric models conventionally leave the baseline survival distribution unspecified but assume a parametric form for how covariates modify this distribution. These semi-parametric models result in complex likelihoods and require assumptions such as the proportional hazards assumption of Cox (1972), or numerical approximations of the likelihood. Such approaches have been echoed in the machine learning community using neural networks (Katzman et al., 2018; Nagpal et al., 2021c). The increased modelling flexibility provided by these can lead to improved predictive performance.

Nonetheless, neural networks approaches have continued to make similar parametric assumptions to obtain closed-form tractable likelihoods (Katzman et al., 2018; Nagpal et al., 2021b), or used numerical approximations and discretization of the timescale to a finite number of time intervals for computational efficiency (Lee et al., 2018). The improved performance compared to non-neural approaches justified the use of these methods but might result in sub-optimal modelling. Additionally, they might exacerbate the interpretability issue of neural networks: the optimisation leads to modelling well the wrong assumption instead of sub-optimal learning of the true distribution. Therefore, any interpretation of the weights might be misleading. This problem limits their applicability in the medical domain for which population-level survival profiles would provide a better understanding of risk and disease.

In this work, we introduce Neural Survival Clustering (NSC): a fully neural approach that models the cumulative hazard function as a mixture of neural networks. Each component models an unconstrained distribution that reflects a survival cluster in the studied population. Individual survival distributions are obtained as a weighted combination of the population-level distributions. These weights are obtained through an assignment network. We show that this method benefits from better interpretability and group discovery compared to existing methods.

This paper first explores the related literature before introducing our proposed model. Next, applications to a synthetic and two real-world datasets demonstrate the effectiveness and interpretability of our approach.

## 2 Related work

The clinical literature traditionally relies on Cox proportional hazards models (Cox, 1972) to model survival outcomes: a linear combination of covariates *h*(*X*) = *β^T^X* is usually used to model deviations from a population's non-parametric baseline hazard λ_0_ on the log-hazard scale, i.e. *λ*(*t*|*X*) = *λ*_0_(*t*)*e*^*h*(*X*)^ where *λ* is the instantaneous risk of an event conditional on survival until that time (the hazard) and *X*, a vector of covariates. This model assumes proportionality between the baseline and the individuals' evolutions. However, this assumption rarely holds in medical applications (Stablein et al., 1981) and extensions have been developed to allow more flexibility, such as stratified group baselines and covariate interactions.

These semi-parametric approaches have been extended to model more complex relationships between covariates and survival. **DeepSurv** (Katzman et al., 2018) extends the Cox Model with non-linear covariate interactions, i.e. *h* is a non-linear function of the covariates, such as the output of a neural network. The neural network's training maximises the model's partial log-likelihood as in traditional Cox models. However, this approach relies on the same proportional hazards assumption. To overcome this issue, **DeepHit** (Lee et al., 2018, 2019) divides the timescale into discrete intervals. The task becomes similar to a classification in which each outcome is a binary variable reflecting if the patient survived within a specific time interval. As a non-parametric model, this approach offers better discriminative performances when the underlying survival distribution is unknown. This model benefits from being effortlessly extendable to competing risks but suffers from its discretisation that limits its applicability.

Another approach consisting of a time discretisation is modelling the hazard as constant on discrete intervals: Rava and Bradic (2020) modelled the problem as step-wise additive hazard functions. Other methods have been explored to avoid assumptions on the survival function: Bender et al. (2021) proposed a general framework for survival analysis by considering the intensity function as an exponential of a non-linear function. This form creates a parallel with Poisson regression and then can leverage any regression model. This approach discretises the prediction horizon to obtain a piece-wise exponential function. In its limit, an infinite discretization of the survival modelling leads to an ordinary differential equation (ODE) which is the approach adopted in Tang et al. (2020). This approach results in an assumption-free model that can maximise the exact likelihood but relies on an ODE solver. Closer to our work, Chilinski and Silva (2020); Omi et al. (2019); Rindt et al. (2021) described another neural network that does not approximate the likelihood while avoiding the computational burden of ODE. The authors propose to model the cumulative intensity function through a monotonic neural network, and leverage automatic differentiation to derive the exact likelihood.

Models have also been developed to leverage parametric distributions while allowing more flexibility. Nagpal et al. (2021a,b) proposes Deep Survival Machine (**DSM**), a mixture of Weibull distributions for predicting the survival of an individual. Parameters of the Weibulls and individual mixture weights are jointly learnt through a deep neural network. However, each component deviates from a population mean through the use of a neural network modelling individual effects.

The previously described approaches have extended survival modelling to complex non-linear dependencies on covariates, improving performance at the cost of interpretability. Discriminative performance is essential for applicability but high stake applications require a better understanding of the survival outcome. For instance, current medical practice relies on identifying groups at different risks to adapt treatment. Models performing sub grouping therefore enhance interpretability and allow personalised treatment (Collins and Varmus, 2015).

Survival clustering has been explored to tackle this issue in three different ways. First, as post-processing: a survival model is fitted to the population and the identified predictive covariates are used for clustering. For instance, Gaynor and Bair (2017); Bair et al. (2004) model survival using a Cox model and applied a K-Means clustering with a weighted distance. Xia et al. (2019) extracts the embedding obtained through a deep learning survival model to cluster the population. Nonetheless, clustering on covariates might not be consistent with outcomes (Bair et al., 2004; Gaynor and Bair, 2017). Second, as an objective in itself: data are clustered given the outcome by maximising the divergence between clusters' survival distributions (Mouli et al., 2019). Finally, as a joint optimisation: both clustering and survival objectives are jointly maximised as in the Bayesian profile regression (Chapfuwa et al., 2020; Liverani et al., 2021) or in Manduchi et al. (2021). Similarly, Nagpal et al. (2021c) explores a mixture of Cox regression with group baselines in which individual covariates allow deviation from the Breslow estimator of the cumulative hazards. Each cluster assumes proportional hazards and the semi-parametric approach requires an expectation-maximisation (EM) optimisation. Direct joint optimisation should be preferred as multistage optimisation and EM approach might lead to suboptimal solution and slow convergence (McLachlan and Krishnan, 2007).

Our work is part of this third family with end to end optimisation. The proposed approach consists of a mixture of neural networks modelling non-parametric distributions of the cumulative hazard function. Each individual survival distribution is a combination of these distributions. This method leverages neural networks to obtain unconstrained cluster distributions while maximising the likelihood of the observed data. This results in a more interpretable neural network that does not rely on the assumptions made by the previous models.

## 3 Proposed Approach

### 3.1 Notation

We aim to model the survival outcome of a given population of the form {*x_i_*, *t_i_*, *d_i_*}_*i*_ where *x*_i_ is a vector of the observed covariates for patient *i, t_i_* ∈ ℝ^+^ is the last time the patient was present in the study, and *d_i_* represents the cause of end of follow-up. We assume non-informative censoring i.e. if *d_i_* = 0, the patient is right-censored for a cause uncorrelated with the outcomes of interest, otherwise an event of interest was observed. In the remainder of this paper, we use “censored” to mean “right-censored”. However, the model can easily be extended to left censoring.

### 3.2 Model

Using a mixture of distributions for the hazard function has led to improved discriminative and calibration performances (Lee et al., 2019; Nagpal et al., 2021b). Previously described mixture models have focused on improving individual performances. These approaches do not enhance group interpretability as the baseline distributions are adjusted for individual characteristics or directly depend on their covariates (Nagpal et al., 2021b,c).

We propose a novel architecture with input *x*, the covariate vector, and the time of prediction, *t*, and with output Λ(*t, x*), the cumulative hazard at time *t*. Each neural network *k* in the mixture outputs Λ*_k_* (*t*), which is defined as the integral of the instantaneous hazard from the time origin until the time *t* at which to evaluate the function. Its input consists of time *t* and a set of latent weights *l_k_*, learnt during training. Each component, therefore, represents the survival distribution of the *k*^th^ cluster and *does not* directly depend on input data, i.e. *x* is not an input of the *k*^th^ cluster.

As integral functions of a positive hazard function, these neural networks need to return a positive value, monotone over time. Chilinski and Silva (2020) introduces monotone neural network for density estimation by enforcing neural network to have positive weights. Omi et al. (2019) applies the non-smooth absolute function to ensure positive weights. We propose to use the log space or square function as in Rindt et al. (2021). This alternative guarantees the derivative's existence. These weights' updates avoid complex optimisation while ensuring the desired property.

Finally, the additional constraint of being null at time *t* = 0 for the cumulative hazard must be enforced. Therefore, the neural network value at the origin time is subtracted from each component. This ensures that each component returns the well defined Λ_*k*_. While the optimisation should enforce this constraint to reach optimal likelihood, its enforcement speeds up convergence and ensures stability and identifiability compared to previous methods (Omi et al., 2019; Rindt et al., 2021).

An individual survival function is then a weighted sum of these neural distributions as follows:

(1) $$\begin{array}{l} S\left(t|x\right)={𝔼}_{Z}\left[\mathbb{P}\left(T\geq t|x,z\right)\right]\\ \;=\underset{k}{\sum }\mathbb{P}\left(z=k|x\right)\mathbb{P}\left(T\geq t|z=k\right)\\ \;=\underset{k}{\sum }\mathbb{P}\left(z=k|x\right)e^{-\Lambda k\left(t\right)} \end{array}$$

in which *z* is the assigned cluster for the data *x*.

This assignment *z* is obtained through an additional neural network which outputs the probability vector *α* of belonging to each components, in which

$$\alpha_{k}\left(x\right)=\mathbb{P}\left(z=k|x\right)$$

**Figure 1:**
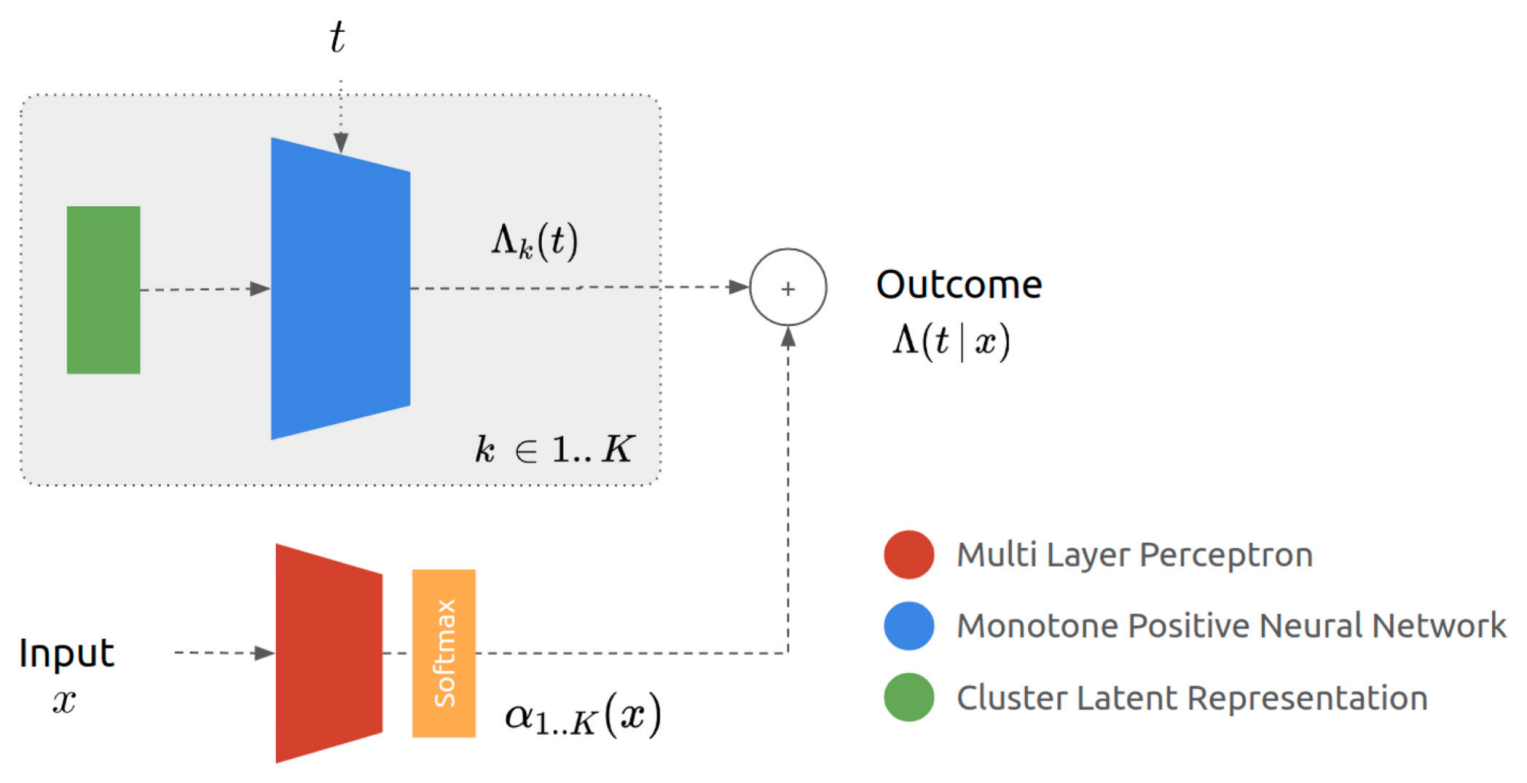
Neural Survival Clustering Architecture.

Figure 1 describes the proposed model. A first multi layer perceptron with inter-layer dropout estimates the mixture weights *α*_1_.._*K*_ with a Softmax to ensure that their summation is equal to one. This assignment neural network leverages the individual data to allocate each point to a cluster. Each component of the mixture of networks takes the time t and the learnt latent representation *l_k_* as inputs to predict the cluster-specific cumulative hazard Λ_*k*_(*t*). Finally, the survival function estimate is obtained as the weighted sum of the components as shown in equation (1). Note that one could consider a unique monotone neural network with a *K*-dimension output to scale to larger number of clusters.

### 3.3 Training Loss

The model is trained by maximising the survival likelihood. Our approach leverages the automatic differentiation used to train neural networks to compute the exact likelihood at no additional computational cost (Omi et al., 2019; Rindt et al., 2021). In our setting, each component *k* computes: *t, l_k_* → Λ_*k*_ (*t*) with *l_k_,* the latent cluster representation and Λ_*k*_, the cumulative hazard function for this *k*^th^ component, i.e. $\Lambda_{k}(t)=\int_{0}^{t}\lambda_{k}(u)du.$ Using automatic differentiation, one obtains the instantaneous hazard function *λk*(*t*).

Focusing on the set of uncensored patients *U*, the likelihood contribution of the observation (*x_i_,t_i_*)_*i*∈*U*_ is the probability of surviving until *t_i_* i.e. *S_k_* (*t_i_*) = *e*^−Λ*k* (*t_i_*)^ multiplied by the instantaneous hazard of observing an event at *t_i_* i.e. *λ_k_* (*t_i_*). This leads to the log likelihood contribution for the set *U*:

(2) $$l_{mix}^{U}=\sum_{i\in \text{U}}\text{log}\;\sum_{k}\alpha_{k}\left(x_{i}\right)\lambda_{k}\left(t_{i}\right)e^{-\Lambda_{k}\left(t_{i}\right)}$$

Similarly, the log likelihood contribution for the set of censored patients *C* consists of the probability of surviving up to the censoring time, and can be computed as follows

(3) $$l_{mix}^{C}=\sum_{i\in \text{C}}\text{log}\;\sum_{k}\alpha_{k}\left(x_{i}\right)e^{-\Lambda k\left(t_{i}\right)}$$

The final model is trained by maximising the log likelihood obtained by summing (2) and (3)

(4) $$l_{mix}=l_{mix}^{C}+l_{mix}^{U}$$

## 4 Experiments

### 4.1 Datasets Description

Following a similar experiment setting and pre-processing as in Nagpal et al. (2021b), we present results on the three following single-event and single-risk datasets:

- METABRIC (Curtis et al., 2012) with 1,904 patients presenting 9 genetics and clinical covariates. 57.9% of the population died from breast cancer.
- SUPPORT (Knaus et al., 1995) consisting of 9,105 patients with 30 demographic and medical history covariates. 68.1% of the cohort died during the 180-day observation period.
- Synthetic (Kvamme et al., 2019) with 25,000 synthetic patients with 3 covariates following a non-linear non-proportional hazard. The censoring rate is 34.5%.

### 4.2 Benchmark Models

For predictive performance comparisons, our method: Neural Survival Clustering (NSC), was compared to a Cox Proportional Hazards model CoxPH (Cox, 1972) which expresses the hazard as

$$\lambda \left(t|x\right)=\lambda_{0}\left(t\right)e^{\beta^{T}\;x}$$

with *λ*_0_(*t*) the unspecified baseline hazard and *β*, the learnt vector of coefficients modelling the covariates' effect on survival. Its deep learning extension **DeepSurv** (Katzman et al., 2018), which leverages a neural network to estimate the covariate effect, was also used for comparison. Moreover, the performance of the monotone survival neural network **SuMo-net** (Rindt et al., 2021) was also compared, as our work uses a similar network for the distribution modelling. Additionally, we analysed the performance of **Deep-Hit** (Lee et al., 2018), which discretizes the survival horizon to train the model as a discrete classification task. Finally, a mixture of Weibull distributions conditioned on a deep representation of the covariates, known as Deep Survival Machine (**DSM** Nagpal et al. (2021b)), was evaluated.

For population clustering, we compare our model to a mixture of Cox models known as Deep Cox Mixture (**DCM** Nagpal et al. (2021c)). While this method allows individual flexibility as each patient can deviate from a non-parametric cluster baseline, it relies on expectation-maximisation iterations and Breslow estimators that might respectively lead to sub-optimal modelling and overfitting. As a final clustering baseline, we considered a Cox-Weighted K-Means **(CWKM)** in which the covariates are divided using a K-means algorithm with an Euclidean distance weighted by the Cox regression and a Kaplan-Meier estimator to estimate the survival distribution for each group.

### 4.3 Experimental Settings

The experiments consist of a 5-fold cross-validation with identical splits for every model. Our proposed approach was fitted on 1000 epochs with hyper-parameters selected over 100 random iterations. The random search used the following grid: learning rate (0.001 or 0.0001), batch size (100 or 250), number of layers for both mixture weights and survival neural networks (1, 2, 3) with number of nodes (50 or 100), number of components for the mixture (〚2, 5〛) and size of the latent cluster representation (10, 50, 100). Adam optimiser (Kingma and Ba, 2015) was used. Finally, *Tanh* activation function was used to ensure the existence of the cumulative intensity's derivative.

The parameter search for all other methods used a similar grid (when appropriate). Additionally, following (Nagpal et al., 2021b), we optimised DSM over the type of distributions (LogNormal or Weibull) and used 10,000 warming epochs. Four intervals were used for DeepHit to discretise the timescale. These splits reflect the evaluation at 0.25, 0.5 and 0.75 quantiles. The training procedure relied on an early stopping criterion on 10% of the training split using the negative log-likelihood loss.

### 4.4 Evaluation metrics

Survival performances were measured using time-dependent Brier score (Graf et al., 1999) and cumulative time-dependent C Index (Hung and Chiang, 2010) at the dataset-specific 0.25, 0.5 and 0.75 quantiles of the uncensored population event times, and averaged over the 5-fold cross-validation. Means and standard deviations are reported.

Table 1 reports the percentage of patients experiencing temporal censoring and observed outcomes of the different datasets at the 0.25, 0.5 and 0.75 quartiles of observed events in the population used for performance evaluation.

Time dependent Brier score was used to measure models' calibration in the presence of right censored data. It is defined at time *t* as:

$$\begin{array}{l} \text{BS}\left(t\right)=\frac{1}{n}\sum_{i}[\omega \left(t_{i}\right){𝟙}_{i\in U\wedge t_{i}\leq t}\hat{S}{\left(t|x_{i}\right)}^{2}\\ \;+\omega \left(t\right){𝟙}_{t_{i}>t}{\left(1-\hat{S}\left(t|x_{i}\right)\right)}^{2}] \end{array}$$

with 𝟙, the indicator function, $\hat{S}\left(t|x\right)$, the predicted survival probability at time *t* and *ω*(*t*), the Kaplan-Meier estimate of the inverse probability of censoring weight.

The time-dependent C index is a generalisation of ROC-AUC to survival labels with right censoring. It captures the discriminative performance of a model by measuring the ordering of the survival predictions:

$$\text{C}\;\text{Index}\left(t\right)=\frac{\sum_{i,j}\omega \left(t_{i}\right){𝟙}_{\left(t_{i}\leq t\right)\wedge \left(t<t_{j}\right)\wedge \left(\hat{S}\left(t|x_{j}\right)>\hat{S}\left(t|x_{i}\right)\right)}}{\left[\sum_{k}{𝟙}_{t_{k}>t}\right]\left[\sum_{k}\omega \left(t_{k}\right){𝟙}_{t_{k}\leq t}\right]}$$

## 5 Results

### 5.1 Performance

Table 2 presents the time-dependent C index and Brier score performance of the different models.

On METABRIC, the proposed approach (NSC) consistently outperforms DCM by a large margin and competes with state-of-the-art deep learning approaches. This advantage might result from the proportional hazards assumption and the sub-optimal expectation-maximisation used by DCM. Note that the competitive advantage of neural network approaches fades at larger time horizons, with a decreasing margin between the Cox model and the best performing models. DeepHit exemplifies this issue as it suffers from less populated horizons. Lastly, leveraging the non-linear relation between covariates provides an edge as shown by the difference between CoxPH and DeepSurv. These results confirm the following observations made in the literature (Wang et al., 2019; Lee et al., 2019): non-parametric models present superior discriminative performance when the survival distribution is unknown or misspecified and, more complex approaches' performance suffers from less populated time horizons.

Identical observations are echoed for the SUPPORT dataset for which the proposed approach offers a significant improvement compared to state-of-theart models. The absence of censored patients and the potential presence of groups (Knaus et al., 1995) might explain this advantage. One can note that DCM presents more competitive results in this example as it might have reached a more stable solution. Lastly, SuMo-net presents similar performance to our model as it relies on a similar structure. Nonetheless, our approach has an interpretability edge by extracting population phenotypes that do not directly rely on the input covariates.

Finally, the Synthetic experiment shows the limit of the proposed method that does not allow the distributions to directly depend on the input data. This explains the competitive advantage of SuMo-net, DeepHit and DSM that model the survival outcome as a non-linear transformation of the covariates. Nonetheless, the existence of phenotypes in real-world medical datasets is better leveraged by our proposed method which results in higher interpretability.

From these experiments one can make the three following conclusions:

- While our approach does not aim to maximise discriminative performances but to discover clusters, it nonetheless challenges other state-of-the-art methods.
- Our method identifies survival distributions aligned with the observed outcome.
- The unconstrained family of survival distributions learnt by our method allows more flexibility compared to DSM and DCM, despite not relying on input covariates.

### 5.2 Clustering

**Figure 2:**
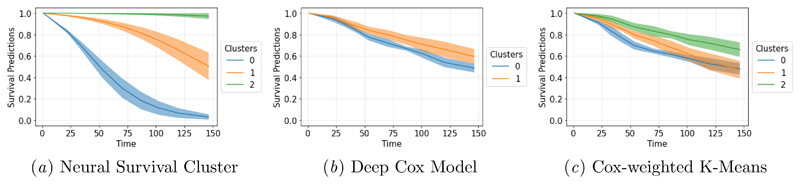
Survival clusters observed in the METABRIC dataset.

The proposed approach aims to provide new insights into the survival distributions present in the data. To demonstrate the capacity of the model to identify groups, we further study the METABRIC results. In this analysis, the number of clusters was selected by an elbow rule on the negative log-likelihood with a fixed number of components (See Figure 3 in the Appendix). Then, the cross-validation was re-run with the selected number of components. Presented in Figure 2 are the average clusters obtained on the METABRIC over the 5-fold test sets. Three main conclusions can be made from this analysis.

First, the family of survival distribution is unconstrained as monotone neural networks are universal approximators (Lang, 2005). This flexibility allows for the recovery of the population clusters despite differences in survival distributions. In this example, one can note how distinguishable are the identified baseline distributions. Additionally, the narrowness of the 95% confidence bands shows the algorithm's consistency over the 5-fold cross-validation, validating the stability of these three clusters in the population.

Second, as further validation of the obtained distributions, every point was assigned to one cluster by discrete allocation to the highest estimated cluster probability of *z*. A Kaplan-Meier estimate was then fitted to estimate the median survival time in each group. A log-rank test tested if the survival distributions were significantly distinct at the 5% level of significance. Table 3 summarises the characteristics of the clusters with the average median survival time obtained over the 5-fold cross-validation, the percentage of the study cohort present in each cluster, the proportion of censored patients and the covariates' average values for DCM, NSC and CWKM. While all methods lead to statistically significantly different clusters' survival distribution, NSC identifies a population of long-term survivors with a median life expectancy after diagnosis close to double that of the other groups.

Third, membership to a cluster can be further studied as the obtained survival distributions do not rely on patients' covariates. A permutation of the covariates (Breiman, 2001) on the assignment network's inputs identified age at diagnosis, chemotherapy indicator and ERBB2 gene marker as the most discriminative covariates between groups (See Figure 5 in the Appendix). These covariates were averaged per group in Table 3. This confirms observations made on the improved recovery for younger patients and the increased risk for patients with ERBB2 marker (Curtis et al., 2012) as patients belonging to cluster 0 show higher predominance of this gene marker and shorter life expectancy. However, the permutation approach does not allow formulating causal conclusions. This limitation is underlined by the chemotherapy distribution: the use of chemotherapy might reflect how advanced the condition is but might also be linked to the genetics of the breast cancer as well as patients' preference and other treatment option. Hence, the observation of lower chemotherapy prevalence in clusters 1 and 2 despite longer median survival times in comparison to cluster 0.

## 6 Conclusion

In this paper, we propose a non-parametric survival clustering approach that consists of a mixture of survival distributions modelled through monotone neural networks. This work builds upon the previous literature by generalising (Nagpal et al., 2021b,c) to non-parametric distributions, independent of the input data while avoiding assumptions of proportional hazards and sub optimal expectation-maximisation (EM) training. The use of neural distributions as an alternative to the Breslow estimators allows an end-to-end optimisation of the observed likelihood leading to a more reliable optimisation (than EM training) and therefore more stable and interpretable clusters. Our approach remains highly interpretable as the neural networks define cluster distributions at the population-level. The input data are only leveraged to identify membership to the different clusters. This work shows state-of-the-art performance while providing better insight into the survival distributions observed in the population. While a deeper exploration of the model's assignment does not lead to causal conclusions, it opens avenues for further research on potential risk factors. As future work, we aim to automatically discover the optimal number of components, left as a parameter tuning problem in this work.

### Institutional Review Board (IRB)

This research does not require IRB approval as it relies on publicly available datasets from studies previously approved.

## Supplementary Material

Appendix

## Figures and Tables

**Table 1:** Percentages of patients observing an outcomes by the evaluation’s times.

Dataset	Outcome	*q*_0.25_	*q*_0.5_	*q*_0.75_
METABRIC	Censored	2.05	6.83	18.86
	Dead	14.50	28.94	43.43
SUPPORT	Censored	0.00	0.00	0.00
	Dead	16.71	33.96	51.03
Synthetic	Censored	5.46	13.01	20.74
	Risk	16.38	32.77	49.15

**Table 2:** Models’ performance - *Mean (standard deviation) over the 5-fold cross validation with best performance in bold and second best in italic*.

		C Index			Brier Score		
	Model	*q*_0.25_	*q*_0.5_	*q*_0.75_	*q*_0.25_	*q*_0.5_	*q*_0.75_
METABRIC
	**NSC**	*0.700* (0.06)	**0.669** (0.05)	**0.647** (0.04)	**0.117** (0.02)	0.192 (0.02)	**0.222** (0.02)
	DCM	0.552 (0.08)	0.543 (0.09)	0.547 (0.09)	0.125 (0.01)	0.210 (0.01)	0.249 (0.01)
	DSM	**0.701** (0.06)	0.662 (0.04)	*0.642* (0.04)	**0.117** (0.02)	*0.191* (0.02)	**0.222** (0.02)
	SuMo-net	**0.701** (0.06)	*0.667* (0.04)	0.640 (0.03)	*0.118* (0.02)	**0.190** (0.02)	*0.223* (0.02)
	DeepHit	0.680 (0.08)	0.631 (0.05)	0.600 (0.03)	0.120 (0.02)	0.200 (0.02)	0.236 (0.01)
	DeepSurv	0.631 (0.04)	0.633 (0.03)	0.634 (0.04)	0.122 (0.02)	0.197 (0.02)	0.227 (0.02)
	CoxPH	0.630 (0.02)	0.626 (0.02)	0.633 (0.03)	0.121 (0.01)	0.196 (0.01)	*0.223* (0.02)
SUPPORT
	**NSC**	*0.749* (0.01)	**0.713** (0.01)	**0.681** (0.01)	*0.128* (0.01)	**0.189** (0.00)	*0.212* (0.00)
	DCM	0.690 (0.10)	0.663 (0.08)	0.639 (0.06)	0.132 (0.01)	*0.200* (0.02)	0.220 (0.02)
	DSM	0.733 (0.01)	*0.699* (0.01)	0.653 (0.01)	0.136 (0.01)	0.204 (0.01)	0.219 (0.00)
	SuMo-net	**0.754** (0.02)	**0.713** (0.01)	*0.680* (0.01)	**0.124** (0.01)	**0.189** (0.01)	**0.211** (0.00)
	DeepHit	0.736 (0.01)	0.685 (0.01)	0.617 (0.01)	0.134 (0.01)	0.210 (0.00)	0.234 (0.00)
	DeepSurv	0.683 (0.01)	0.665 (0.01)	0.663 (0.01)	0.134 (0.01)	0.201 (0.01)	0.216 (0.00)
	CoxPH	0.683 (0.02)	0.668 (0.01)	0.667 (0.01)	0.135 (0.01)	0.201 (0.01)	0.214 (0.00)
Synthetic
	**NSC**	0.856 (0.01)	0.838 (0.00)	0.802 (0.00)	0.097 (0.00)	0.134 (0.00)	0.131 (0.00)
	DCM	0.850 (0.00)	0.827 (0.00)	0.806 (0.00)	0.095 (0.00)	0.131 (0.00)	0.145 (0.00)
	DSM	0.858 (0.01)	*0.841* (0.00)	**0.827** (0.00)	*0.085* (0.00)	*0.122* (0.00)	0.121 (0.00)
	SuMo-net	**0.861** (0.01)	**0.843** (0.00)	**0.827** (0.01)	**0.084** (0.00)	**0.117** (0.00)	**0.112** (0.00)
	DeepHit	*0.859* (0.01)	0.839 (0.01)	*0.818* (0.01)	0.100 (0.00)	0.153 (0.00)	0.153 (0.00)
	DeepSurv	0.846 (0.01)	0.834 (0.00)	**0.827** (0.00)	0.087 (0.00)	*0.122* (0.00)	*0.116* (0.00)
	CoxPH	0.846 (0.00)	0.821 (0.00)	0.794 (0.00)	0.092 (0.00)	0.134 (0.00)	0.152 (0.00)

**Table 3:** METABRIC - Clusters’ characteristics

Models	Cluster 0						
	Median Survival	Population %	Censored	||	Age At Diagnosis	Chemotherapy	ERBB2
**NSC**	102.22	23.95 %	33.55 %	||	61.20	51.75 %	6.12
DCM	138.97	71.64 %	37.31 %	||	64.10	22.95 %	5.88
CWKM	139.90	19.22 %	49.18 %	||	48.63	99.73 %	6.01
						
	Cluster 1						
	Median Survival	Population %	Censored	||	Age At Diagnosis	Chemotherapy	ERBB2
**NSC**	135.75	45.06 %	33.57 %	||	68.94	0.23 %	5.80
DCM	205.71	28.36 %	54.07 %	||	53.46	15.37 %	5.85
CWKM	125.17	47.69 %	28.41 %	||	72.13	3.41 %	5.84
	Cluster 2						
	Median Survival	Population %	Censored	||	Age At Diagnosis	Chemotherapy	ERBB2
**NSC**	>237.82	30.99 %	61.02 %	||	49.58	26.78 %	5.79
CWKM	230.71	33.09 %	57.62 %	||	52.41	0.00 %	5.84

## Data Availability

This paper uses the publicly available datasets: METABRIC (Curtis et al., 2012), SUPPORT (Knaus et al., 1995) and a synthetic dataset (Kvamme et al., 2019), all available on Github^1^. The code for the proposed model and to replicate our results is also available on Github^2^.
