# Supplementary material for: Neural Survival Clustering: Non-parametric mixture of neural networks for survival clustering
Source: Proc Mach Learn Res. Author manuscript; Available in PMC 2022 Apr 22. (PMC7612649)
Supplement: Appendix [file EMS144308-supplement-Appendix.pdf]

7. Appendix

This appendix provides additional results on the METABRIC cluster analysis.

Figures 3 (resp. 4) presents the log likelihood (resp. C-index) on the METABRIC cross validation test sets for an increasing number of clusters. The red lines intersections identify the elbow number of clusters between 2 and 3 clusters.

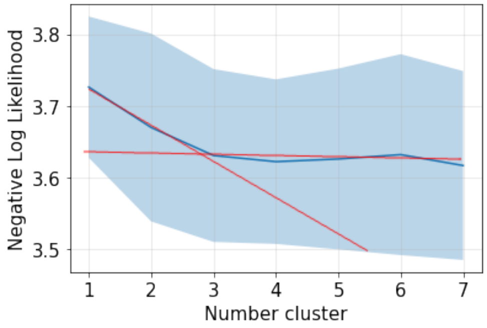

Figure 3: METABRIC - Log likelihood evolution given the number of clusters

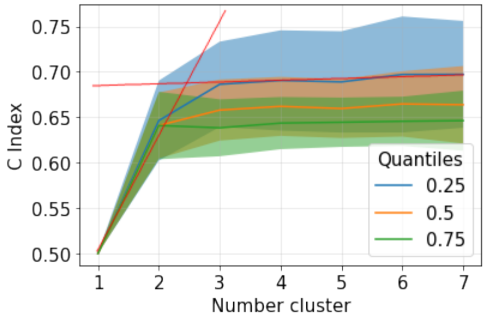

Figure 4: METABRIC - C index evolution given the number of clusters

Figure 5 shows the relative change of the model's likelihood under permutation of the input covariates. This identifies which features impact the model's likelihood the most. As input covariates only influence group membership, this gives an intuition of which features are responsible of this assignment. For comparison, cross validated Cox weights are averaged in Figure 6. The observed differences in feature importance is the results of our approach's non linearity. However, the importance of age at diagnostic and chemotherapy in both analyses underlines the relevance of these features in estimating survival.

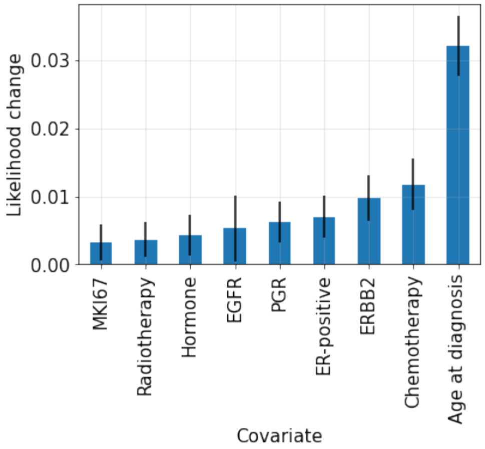

Figure 5: METABRIC - Feature importance obtained through permutation test for NSC with 3 clusters

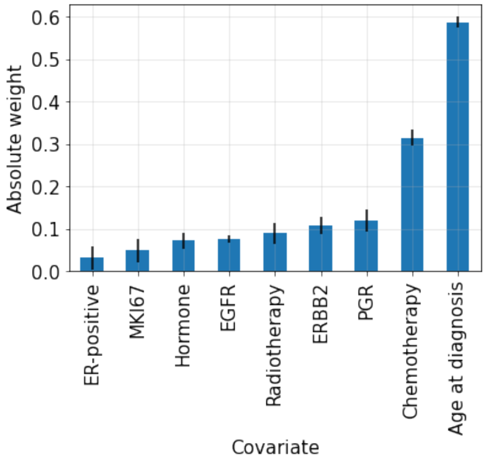

Figure 6: METABRIC - Feature importance for Cox regression
